# A Comparative Proteomic Analysis to Explore the Influencing Factors on Endometritis Using LC-MS/MS

**DOI:** 10.3390/ijms241210018

**Published:** 2023-06-12

**Authors:** Xingcan Jiang, Ziyuan Li, Xiyv Chang, Zhengjie Lian, Aihua Wang, Pengfei Lin, Huatao Chen, Dong Zhou, Keqiong Tang, Yaping Jin

**Affiliations:** 1 Key Laboratory of Animal Biotechnology of the Ministry of Agriculture, College of Veterinary Medicine, Northwest A&F University, Yangling, Xianyang 712100, China; xingcanjiang@nwafu.edu.cn (X.J.);; 2 Department of Clinical Veterinary Medicine, College of Veterinary Medicine, Northwest A&F University, Yangling, Xianyang 712100, China; 3 Department of Preventive Veterinary Medicine, College of Veterinary Medicine, Northwest A&F University, Yangling, Xianyang 712100, China

**Keywords:** endometritis, goat endometrial epithelial cells, LPS, expression profile, label-free proteomics

## Abstract

The inflammatory system activated by uterine infection is associated with decreased fertility. Diseases can be detected in advance by identifying biomarkers of several uterine diseases. Escherichia coli is one of the most frequent bacteria that is involved in pathogenic processes in dairy goats. The purpose of this study was to investigate the effect of endotoxin on protein expression in goat endometrial epithelial cells. In this study, the LC–MS/MS approach was employed to investigate the proteome profile of goat endometrial epithelial cells. A total of 1180 proteins were identified in the goat Endometrial Epithelial Cells and LPS-treated goat Endometrial Epithelial Cell groups, of which, 313 differentially expressed proteins were accurately screened. The proteomic results were independently verified by WB, TEM and IF techniques, and the same conclusion was obtained. To conclude, this model is suitable for the further study of infertility caused by endometrial damage caused by endotoxin. These findings may provide useful information for the prevention and treatment of endometritis.

## 1. Introduction

Uterine diseases, such as hysteritis and endometritis, are common in high-yield dairy goats and lead to economic losses due to a decline in milk production and fecundity [1,2]. Gram-negative bacteria are usually associated with uterine infection in dairy goats [2,3]. Dystocia and placental retention of dairy goats make them vulnerable to uterine diseases because the physical barrier of infection is broken and the immune response is disrupted, so pathogens should be eliminated [2,3].

Successful implantation requires a balance and accurate molecular communication between the embryo and the maternal endometrium [3,4]. Even a small imbalance could negatively affect the dialog between the mother and the embryo necessary for the establishment of pregnancy [4].

According to the diagnosis of endometrial cytology, the prevalence of endometritis in dairy cows is very high [4,5], which increases the reproductive cycle and time costs [5]. Several studies have confirmed that the incidence of endometritis 35–45 days after delivery is very high [5,6], resulting in a significant reduction in the fertility rate of the population [5,6]. Although consistently contaminated or infected in the early postpartum period [6], it is essentially sterile by approximately 28 days postpartum [5,6]. Finally, the characteristics of the relationship between uterine infection and endometritis can reveal the mechanism by which endometritis prolongs pregnancy intervals and even leads to infertility [7,8].

## 2. Results

### 2.1. Identification and Hierarchical Clustering Heatmap

**Figure 1:**
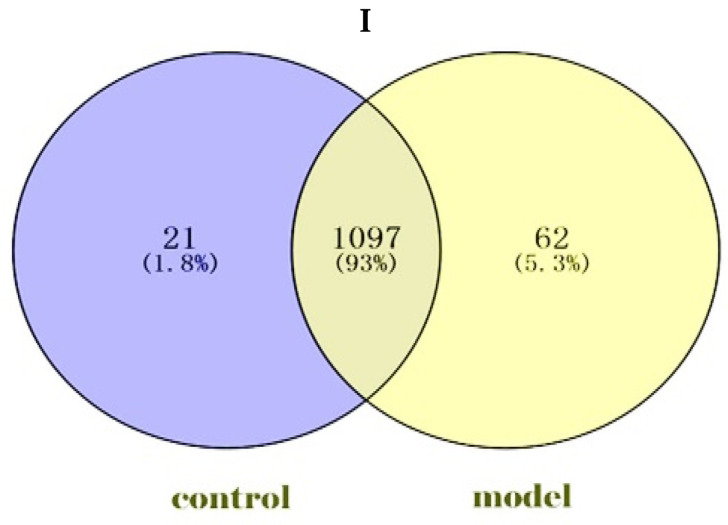

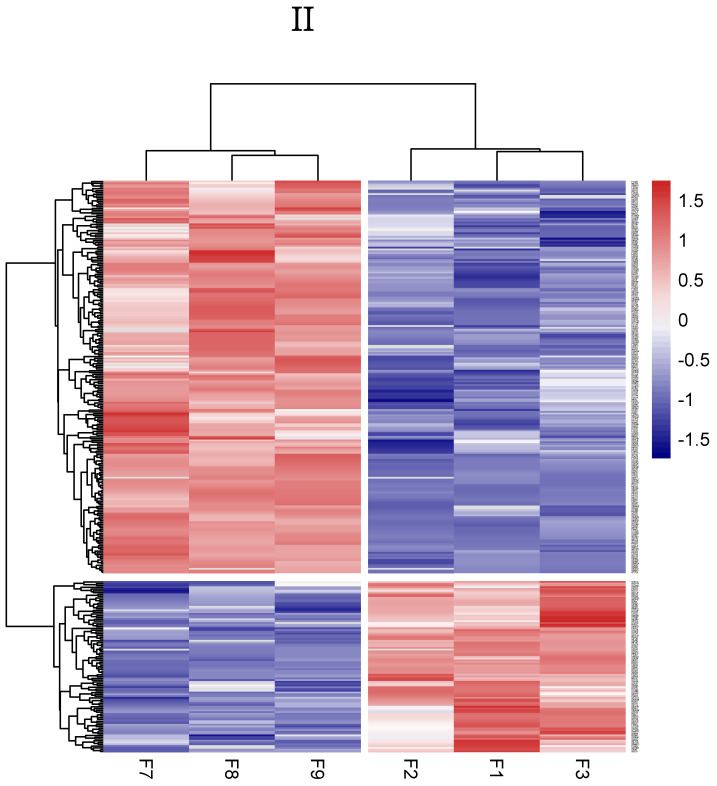
Identification and hierarchical clustering heatmap. (**I**): Screening of Expressed Proteins. (control: goat Endometrial Epithelial Cells; model: LPS-treated goat Endometrial Epithelial Cells. Venn diagram). (**II**): Cluster heatmap of the expression intensity of the differentially expressed proteins (DEPs) from gEECs and LPS-treated gEEC groups. Red indicates increased abundance; blue indicates decreased abundance. Each row shows the relative abundance of a protein in the six different groups, and each column shows the relative abundance of DEPs in each experimental group. The numbers F1, F2, F3, F7, F8 and F9 indicate the individual replicates in each group.

We identified a total of 1180 proteins in the goat Endometrial Epithelial Cells (gEECs) and LPS-treated goat Endometrial Epithelial Cell groups. Among them, 1097 proteins were common in both groups, and 21 and 62 proteins were unique to the gEECs and LPS-treated gEEC groups, respectively (Figure 1I). At the same time, 313 differentially expressed proteins were screened by *p*-value (Supplementary File S1). For the gEECs and LPS-treated gEEC groups, protein clustering can be seen, as in Figure 1II. The hierarchical clustering analysis of the two groups showed that F1 and F3 and F8 and F9 were clustered together at first, followed by F2 and F7. The red color showed the up-regulated expression, and the blue color represented the down-regulated expression. The color from red/blue to white represented the ratio from large to small. The cluster analysis of different proteins shows that the cluster map can be divided into several parts. The sensitivity of these proteins to inflammation is particularly important. The proteins clustered together indicate a consistent response to inflammation, see Supplementary File S1.

### 2.2. Gene Ontology (GO) Functional Category Analysis

**Figure 2:**
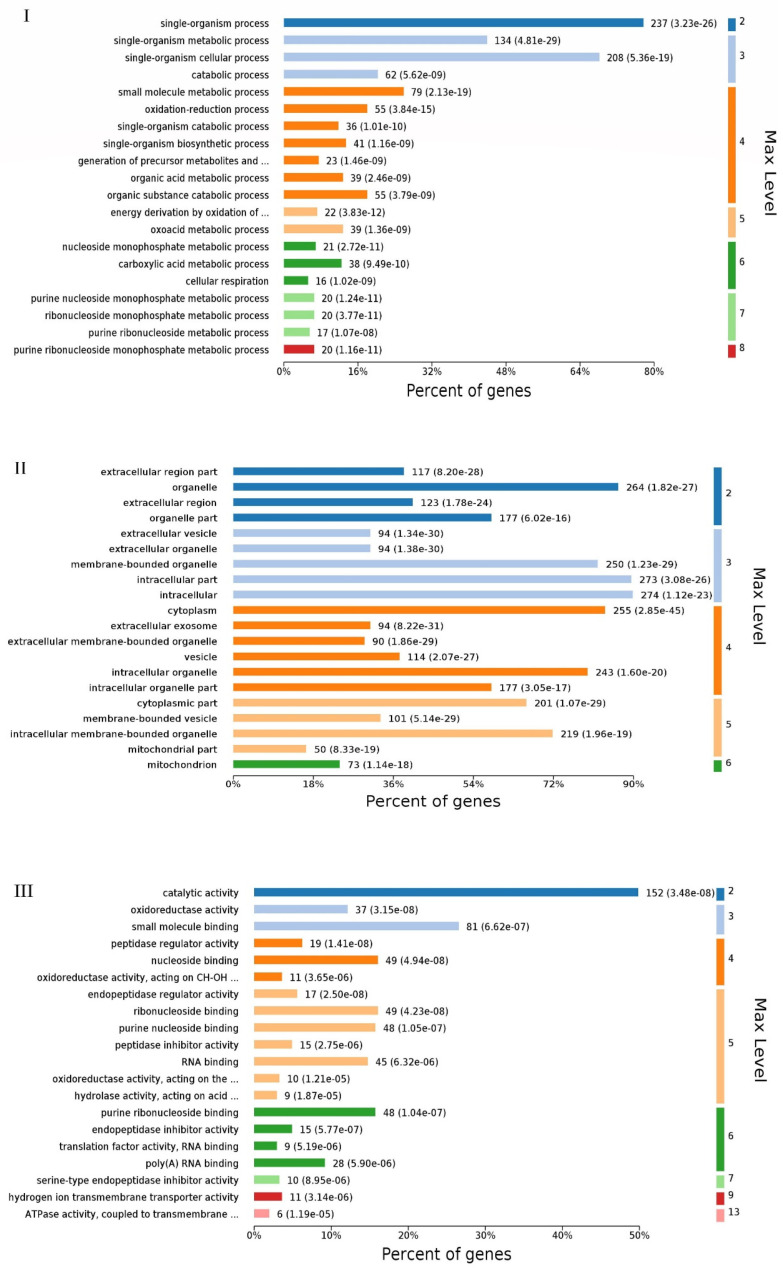
Gene ontology (GO) analysis. (**I**): biological process (BP), (**II**): cellular components (CC) and (**III**): molecular function (MF). Gene ontology classification of differentially expressed proteins based on biological process, cellular components and molecular function. Vertical coordinates indicate different functional annotations, and the abscissas indicate the percentage of proteins enriched in the corresponding annotation among all DEPs.

When analyzing differentially expressed proteins, there were significant differences in biological process (Figure 2I), such as the single-organism metabolic process, single-organism process and so on. Although they were insignificant, there are still differences in differentially expressed proteins, such as the regulation of membrane potential, regulation of protein targeting, response to estrogen, necroptotic process, maturation of LSU-rRNA, response to oxidative stress, organophosphate biosynthetic process, mitophagy, mitochondrion disassembly and so on.

When analyzing differentially expressed proteins, there were significant differences in cellular components (Figure 2II), such as the extracellular vesicles, extracellular organelles, cytoplasmic components, membrane-bound organelles, extracellular membrane-bound organelles, membrane-bound vesicles, extracellular region components, intracellular organelles, intracellular membrane-bound organelles and so on. Although they were insignificant, there are still differences in differentially expressed proteins, such as the nascent polypeptide-associated complex, endoplasmic reticulum-Golgi intermediate compartment, late endosome membrane, early endosome, lytic vacuole membrane, eukaryotic translation elongation factor 1 complex, Cul7-RING ubiquitin ligase complex, lysosomal membrane, late endosome, Golgi membrane, COP9 signalosome, plasma membrane region, vacuolar components, recycling endosome membrane, lysosomal lumen and so on.

When analyzing differentially expressed proteins, there were significant differences in molecular function (Figure 2III), such as peptidase regulator activity, endopeptidase regulator activity, oxidoreductase activity, catalytic activity, hydrogen, transmembrane movement of substances, ATPase coupled, anion binding, protein complex binding, hydrogen-exporting ATPase activity, nucleotide binding, nucleoside phosphate binding, ATPase activity, coupling, NAD(P)H oxidoreductase activity, NADH dehydrogenase activity and so on. Although they were insignificant, there are still differences in differentially expressed proteins, such as, helicase activity; quinone or similar compounds as acceptors; iron-responsive element binding; glucosylceramidase activity; 6-phosphofructokinase activity; translation release factor activity; codon specific, calmodulin-dependent protein phosphatase activity; beta-N-acetylhexosaminidase activity; sequence-specific mRNA binding; transferase activity; transferring of aldehyde and ketonic groups and so on.

### 2.3. Pathway Analysis

**Figure 3:**
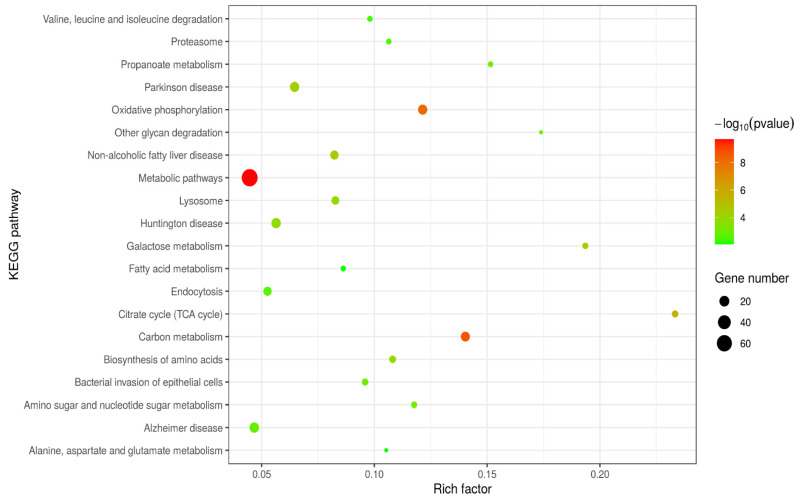
Kyoto Encyclopedia of Genes and Genomes (KEGG) enrichment analysis of the differentially expressed proteins (DEPs). The size of the circle represents the degree of enrichment. The closer the colour is to red, the smaller the *p*-value, and the greater the degree of significance. DEPs: differentially expressed proteins.

We performed Kyoto Encyclopedia of Genes and Genomes (KEGG) pathway analysis on differentially expressed proteins (DEPs), as shown in Figure 3. We obtained a total of 199 KEGG pathways, see Supplementary File S2.

When comparing the the gEECs and LPS-treated gEEC groups, metabolic pathways, carbon metabolism pathways, oxidative phosphorylation pathways, citrate cycle (TCA cycle) pathways, and endocytosis pathways were significantly different. Phagosome pathways were also significantly different. The lysine degradation pathway, one carbon pool by folate pathway, 2-oxocarboxylic acid metabolism pathway, purine metabolism pathway, glucagon signaling pathway and so on were different as well, but the difference was not significant.

### 2.4. PPI Network and Module Analysis of the Identified Proteins

We built PPI networks of proteins identified in the gEECs and LPS-treated gEEC groups.

**Figure 4:**
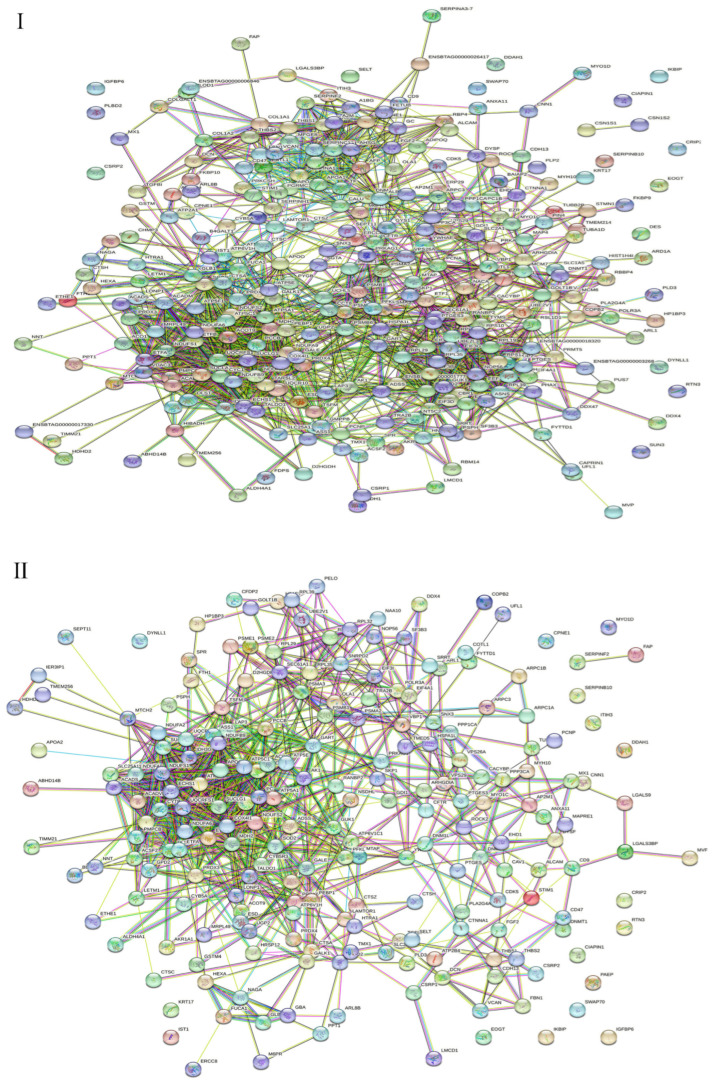

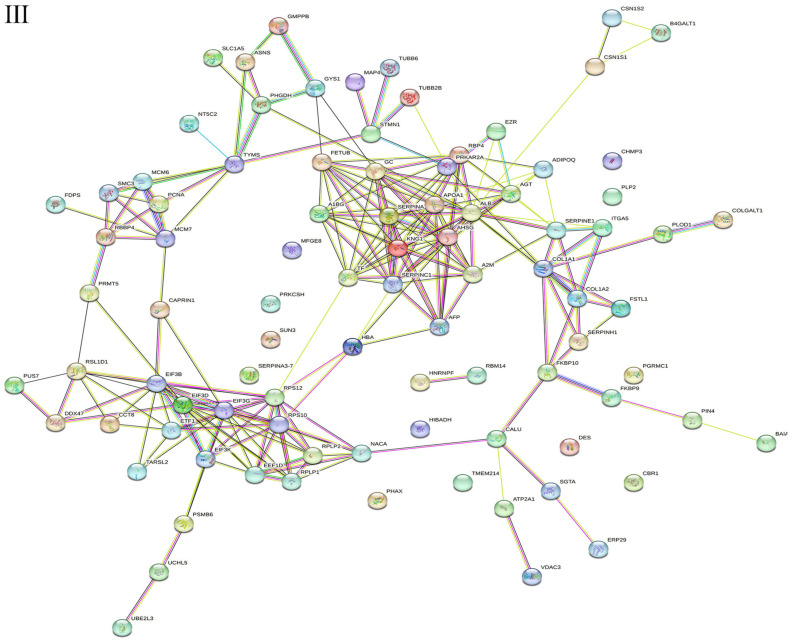
Protein–protein interaction (PPI) network of proteins identified in gEECs and LPS-treated gEEC groups. (**I**): differentially expressed proteins (DEPs). (**II**): up-regulated proteins. (**III**): down-regulated proteins. Protein–protein interactions (Evidence Mode) of dysregulated proteins were predicted by STRING. Each circle node represents 1 protein, the connection between node and node represents the interaction between 2 proteins, and lines with different colors indicate different interaction types. Colored nodes: query proteins and first shell of interactors, white nodes: second shell of interactors, filled nodes: some 3D structure is known or predicted. The network nodes represent proteins from the goat database, and the lines are the predicted functional annotations (red line—the presence of fusion evidence; green line—neighborhood evidence; blue line—cooccurrence evidence; purple line—experimental evidence; yellow line—text mining evidence; light-blue line—database evidence; black line—co-expression evidence).

All significantly different proteins in the gEECs and LPS-treated gEEC groups were analyzed by PPI. All 313 differentially expressed proteins were included in the protein interaction network of up-and down-regulated proteins following LPS stimulation of goat Endometrial Epithelial Cells (Figure 4I). Among of them were 218 up-regulated proteins (Figure 4II) and 95 down-regulated proteins (Figure 4III). The PPI diagram shows that many kinds of differential proteins are related to each other, and the protein–protein interaction (PPI) network is highly complex. Network nodes represent proteins, while edges represent protein–protein associations which were already known (light blue and purple), or predicted (other colors) by String analysis.

From the PPI map, we can see interactions between the OLA1 and A2M; FETUB and SERPINF2; ALB and VCAN, TF and CALU, and TF and RBP4 proteins, and so on. From the PPI map, we can see interactions between the PSMB6 and UCHL5, PSMB6 and NDUFA2, ETFB and ETFA, HIBADH and ALDH4A1, and HIBADH and ABHD14B proteins, and so on. From the PPI map, we can see interactions between the CBR1 and AKR1A1, CBR1 and SPR; CBR1 and GART, DDX47 and PUS7, DDX47 and NOP56, PSMA3 and PSMA2, and PSMA3 and PSMB1 proteins, see Supplementary Files S1 and S2.

### 2.5. Transmission Electron Microscopy (TEM)

**Figure 5:**
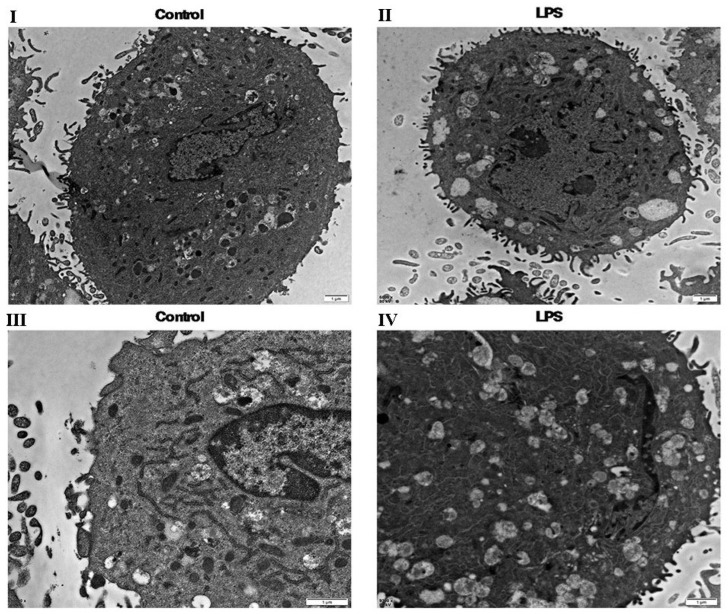
Transmission Electron Microscopy (TEM). (**I**): control group. (**II**): LPS-treated group. (**III**): control group. (**IV**): LPS-treated group. TEM analysis highlights the morphological differences in the gEECs and LPS-treated gEEC groups, (**I**,**II**) (ruler: 1 μm, control group), (**III**,**IV**) (ruler: 1 μm, LPS-treated group).

KEGG analysis showed that the lysosome, proteasome and endocytosis pathways were significantly different between the gEECs and LPS-treated gEEC groups. The TEM results clearly show that the number of autophagic vesicles formed by late lysosomes in the LPS-treated gEECs group is much greater than that in the gEECs group (Figure 5).

The results of proteomics were consistent with those of transmission electron microscopy. This means that lysosomes, autophagosomes and carbon metabolism are more active in LPS-treated gEECs than gEECs.

### 2.6. qRT-PCR Analysis

**Figure 6:**
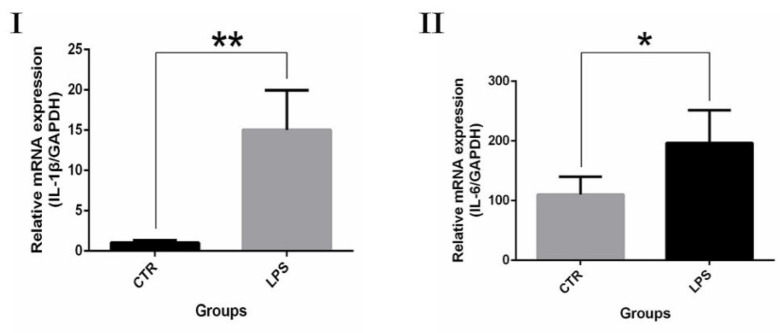
qRT-PCR analysis. (**I**): IL-1β. (**II**): IL-6. The effect of LPS on inflammatory cytokine expression quantified by qRT–PCR after treatment with 3 μg/mL LPS for 24 h. The data are presented as the mean ± SEM from three independent experiments, and bars with different * and ** are significantly different (*p* < 0.05).

Real-time PCR was performed to examine differences in the expression of inflammatory factor in LPS-mediated gEECs inflammatory response. Significantly more IL-1β and IL-6 mRNA was present in the LPS stimulation group compared to the control group (Figure 6). The change in inflammatory variables was conspicuous, proving that LPS were successful in making gEECs become inflammatory.

### 2.7. Western Blot

**Figure 7:**
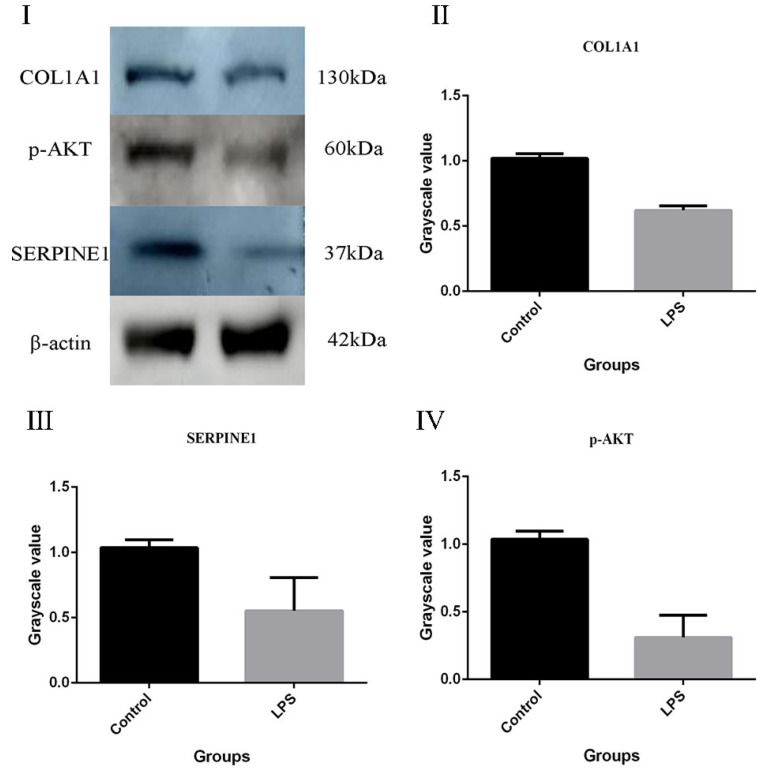
Confirmation of two differentially expressed proteins by Western blot analysis in the gEECs and LPS-treated gEEC groups. (**I**): result of WB. (**II**): COL1A1. (**III**): SERPINE1. (**IV**): p-AKT. β-actin was used as an internal reference to normalize the quantitative data. The data are presented as the mean ± SEM from three independent experiments.

To validate the DEPs identified from the label-free quantitation analysis, proteins with important physiological functions were selected for detection. COL1A1 (Figure 7II) is involved in the structural components of extracellular matrix, platelet-derived growth factor binding, vascular development, the cell response to mechanical stimulation, the collagen-activated tyrosine–kinase receptor signal pathway, the collagen biosynthesis, positive regulation of cell migration, positive regulation of epithelial–mesenchymal transformation, and binding of platelet-derived growth factor. COL1A1 is a type I collagen. It binds calcium ions, which are necessary for its function. The p-AKT (Figure 7IV) has a significant impact on cell survival and growth. Survival factors can suppress apoptosis in a transcription-independent manner by activating the serine/threonine kinase AKT1, which then phosphorylates and inactivates components of the apoptotic machinery. SERINE1 (PAI, Figure 7III) inhibits TMPRSS7. As a PLAU inhibitor, it is involved in cellular and replicative senescence. Equal amounts of proteins from the gEECs were detected with antibodies, as shown in Figure 7. The results showed that the ratios of the selected proteins were consistent with those obtained from the label-free quantitation analysis. In future work, we will conduct an in-depth study of the above proteins.

### 2.8. Immunofluorescence

**Figure 8:**
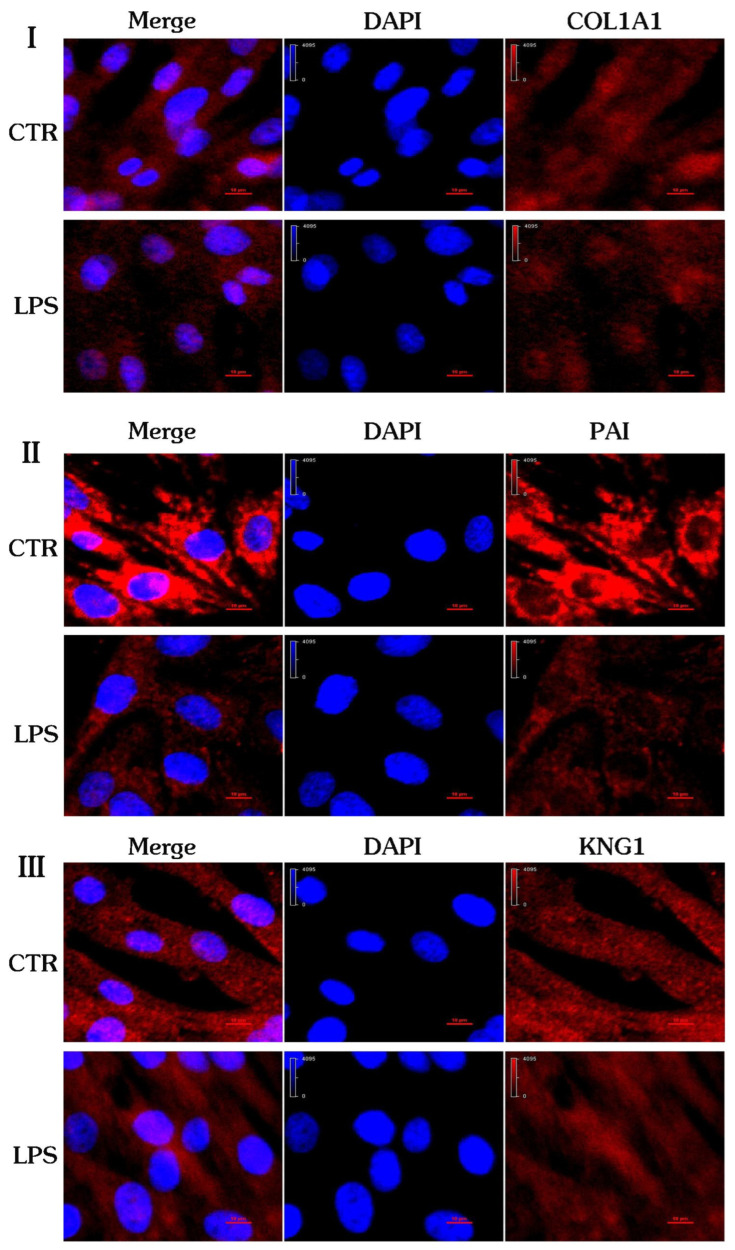
Confocal fluorescence images of the gEECs and LPS-treated gEEC groups. (**I**): COL1A1. (**II**): PAI. (**III**): KNG1.

From Figure 8, it can be seen that the protein localization of COL1A1 (Figure 8I), SERPINE1 (PAI, (Figure 8II)) and KNG1 (Figure 8III) is in the cytoplasm, which is consistent with the results of GO analysis. The differential expression of COL1A1 and SERPINE1 (PAI) and KNG1 indicates that the protein expression profile of the control group and the inflammation group has changed, and it also shows that the biological function of the cells has changed from a side point of view, which may be the main cause of pregnancy failure.

## 3. Discussion

### 3.1. Proteome Quality Difference between gEECs and LPS Treated gEECs Groups

Compared with the gEECs group, the expression of Q6QAT4 was downregulated in the LPS-treated gEECs group. Q6QAT4 is involved in antigen delivery in the immune system. It is also involved in antigen processing and presentation of exogenous protein antigens via MHC class I b, which are TAP-dependent processes. It is closely related to the inflammatory response. RPLP2 expression was downregulated in the LPS-treated gEECs group. RPLP2 participates in the process of protein synthesis and extension and is a structural constituent of ribosomes. This may indicate that the protein synthesis process is abnormal.

### 3.2. The Possible Role of Distinct Proteins Based on GO

When comparing the gEECs group, PRKAR2A expression was downregulated in the LPS-treated gEECs group. PRKAR2A has cAMP-dependent protein–kinase regulator activity and is involved in cAMP and ubiquitin–protein–ligase binding. It participates in the regulation of cAMP-mediated signaling. Compared with the gEECs group, KNG1 gene expression was downregulated in the LPS-treated gEECs group. This study was successfully verified by the immunofluorescence experiment. KNG1 is a mediator of inflammation. It has cysteine-type endopeptidase inhibitor activity. It participates in the inflammatory response and negative regulation of blood coagulation. When comparing the gEECs group, PPT1 expression was upregulated in the LPS-treated gEECs group. PPT1 removes thioester-linked fatty acyl groups. This may indicate that inflammation is related to cellular fat metabolism [5].

### 3.3. The Possible Role of Significantly Different Proteins in Cell Migration, Adhesion and Implantation

When comparing the gEECs group, ITGA5 expression was downregulated in the LPS-treated gEECs group. ITGA5:ITGB1 acts as the receptor of fibrin 1 (FBN1), playing the role of adhesion. When comparing the gEECs group, SERPINE1 expression was downregulated in the LPS-treated gEECs group. As a PLAT inhibitor, SERPINE1is required for fibrinolysis downregulation and is responsible for the controlled degradation of blood clots.

### 3.4. The Possible Role of Different Proteins in Epithelial-Mesenchymal Transformation and Polarization

When comparing the gEECs group, COL1A1 expression was downregulated in the LPS-treated gEECs group. COL1A1 is an extracellular matrix structural constituent. It participates in blood vessel development, the collagen-activated tyrosine kinase receptor signaling pathway. This is consistent with the study of endometrial protein and uterine fluid Wang C and Li T [2,8].

### 3.5. The Possible Role of Significantly Different Proteins in the Process of Apoptosis and the Cell Cycle

When comparing the gEECs group, MCM6 expression was downregulated in the LPS-treated gEECs group. MCM6 acts as a component of the MCM2-7 complex (MCM complex). It combines with ATP and DNA. The biological processes in which it is involved are the cell cycle and DNA replication initiation. When comparing the gEECs group, ANXA11 expression was upregulated in the LPS-treated gEECs group. ANXA11 is involved in the process of mesosome formation and cytokinesis. The biological processes in which it is involved are the cell cycle and cell division. The changes of these proteins are closely related to the changes of endometrial function.

### 3.6. The Possible Role of Significantly Different Proteins in Hormones

When comparing the gEEC group, CYB5R3 expression was upregulated in the LPS-treated gEECs group. The biological processes in which CYB5R3 is involved are cholesterol biosynthesis [9,10]. When comparing the gEECs group, CSN1S2 expression was downregulated in the LPS-treated gEECs group. Casocidin-I inhibits the growth of *E. coli* and *S. carnosus*. The biological processes in which it is involved are the defense response to bacterium, response to dehydroepiandrosterone, response to estradiol, response to growth hormone and response to progesterone.

### 3.7. The Possible Role of Significantly Different Proteins in the Immune System

When comparing the gEECs group, the expression of RBM14 in LPS-treated gEECs group was down-regulated. RBM14 may act as a nuclear receptor coactivator, promoting transcription through other coactivators. It regulates centrosome biogenesis by inhibiting the formation of abnormal centrosomal-protein complexes in the cytoplasm, thus maintaining the integrity of mitotic spindles. It is involved in the regulation of innate immune response mediated by the DNA virus by assembling HDP–RNP complex. HDP–RNP complex is the platform for IRF3 phosphorylation. This suggests that inflammation is closely related to protein phosphorylation.

### 3.8. The Possible Role of Significantly Different Proteins in Stress

When comparing the gEECs group, UFL1 expression was upregulated in the LPS-treated gEECs group. It participates in response to endoplasmic reticulum stress. It participates in ufmylation-dependent reticulophagy and inhibits the unfolded protein response (UPR) via ERN1/IRE1-alpha. When comparing the gEECs group, VBP1 expression was upregulated in the LPS-treated gEECs group. VBP1 binds specifically to cytosolic chaperonin (c-CPN) and transfers target proteins to it. The biological process in which it is involved is protein folding. When comparing the gEECs group, PSMB1 expression was upregulated in the LPS-treated gEECs group. The noncatalytic component of the 20S core proteasome complex is involved in the proteolytic degradation of most intracellular proteins.

### 3.9. The Possible Role of Significantly Different Proteins in Energy Supply

When comparing the gEECs group, the expression of NDUFA9 in the LPS-treated gEECs group was up-regulated. NDUFA9 is a subsidiary subunit of mitochondrial membrane respiratory chain NADH dehydrogenase. Compared with the gEECs group, MDH2 expression was upregulated in the LPS-treated gEECs group. The biological process in which MDH2is involved is the tricarboxylic acid cycle.

## 4. Materials and Methods

### 4.1. Goat Endometrial Epithelial Cells (gEECs) Culture

Trypsin (sequencing grade) was obtained from Promega Corporation (Madison, WI, USA) [7]. A Micro BCA protein assay kit and Pierce C18 tips were obtained from Thermo Fisher Scientific (Rockford, IL, USA) [7,8]. Trifluoroacetic acid, dithiothreitol, iodoacetamide, methanol, acetonitrile, formic acid, and all other chemicals were of analytical grade and obtained from Sigma Aldrich Corporation (St. Louis, MO, USA) [7,8]. All aqueous solutions were prepared using Milli-Q treated water [8].

The hTERT–EECs, which were established and kept in our lab, were in good condition, maintaining normal cellular characters [8,11], such as serum dependence, contact inhibition, chromosome number and morphology, and no suspension growth and tumorigenicity [11]. Epithelial cells were cultured in F-12 medium (Sigma, USA) containing10% Fetal Bovine Serum, 1%Penstrep^®^ (5000 units/mL penicillin/streptomycin) [8,11]. Cells were seeded into a 25 cm [2] ventilation flask and cultured in a water-jacked incubator with 5% CO_2_ at 37 °C [11].

### 4.2. Goat Endometrial Epithelial Cells (gEECs) Challenged with E. coli LPS

Cultured gEECs were challenged with *E. coli* LPS (Dulbecco’s Modified Eagle Medium, DMEM/F12, Hyclone, Logan, UT, USA) with concentrations in the range of those previously reported in vitro studies [11,12]. LPS (0 and 3 μg/mL LPS) was applied to the gEECs for 24 h with three biological repeats per sample [12].

### 4.3. Extraction of Cell Samples

Cellular pellets were solubilized in a buffer with protease inhibitors [12,13]. Briefly, the cells were washed with PBS buffer for 5 min each time, split with 20 μL cleavage buffer, scraped off, and placed on ice [13].

Afterward, the cells were centrifuged at room temperature at 10,000 rpm for 10 min. The precipitate was discarded [13,14]. Protein quantification was performed using a bicinchoninic acid (BCA) Protein Assay quantification kit. All steps are carried out according to the instructions for the reagent [14].

### 4.4. Preparation of Colloidal Particles

The samples were prepared for short-range SDS-PAGE (12%) [14,15]. Gels were stained in colloidal Coomassie blue. After gel staining, each lane was cut into small pieces and subjected to in-gel digestion with SimplyBlue Safe Stain (Invitrogen, Carlsbad, CA, USA) [15]. All the steps were operated in the specified temperature range.

### 4.5. Digestion in Protein Gel

A gel containing 100 μg of proteins was mixed with dithiothreitol (final concentration of 10 mM) and kept at 60 °C for 70 min [15,16]. After the sample was cooled, iodine acetamide with a final concentration of 66 mM was added and reacted away from light at room temperature for 40 min [16].

After that, 200 μL NH_4_HCO_3_ solution (50 mM) was added to the filter and centrifuged at 14,000× *g* for 25 min. Finally, 1.25 μg of trypsin was added and the mixture incubated at 40 °C overnight [16,17]. The digestion procedure was stopped by adding formic acid (10%), and digested peptides were collected by centrifuging at 14,000× *g* for 15 min. After desalting with the C18 tips, the collected peptides were vacuum-dried [17,18]. After trypsin digestion, Ziptip C18 micropipette tips (Millipore) were used to purify the peptides prior to adding 0.1% formic acid for LC-MS/MS analysis.

### 4.6. LC–MS/MS Analysis

We used the Easy-nLC 1200 system coupled with a Orbitrap Fusion Lumos mass spectrometer for LC-MS/MS analysis, according to the method reported previously [17,18,19]. Dried samples were re-suspended with mobile phase A (0.1% formic acid in water) [17,18]. Then, samples were loaded onto a pre-column (100 μm × 2 cm; 5 μm; C18 particles) and separated by a linear-gradient elution with mobile phase B on an analytical column at a flow rate of 300 μL/h [17]. Analysis time: 0 min (5% phase B), 1 min (12%), 29 min (27%), 44 min (38%), 49 min (55%), 50 min (100%), and 60 min (100% phase B) [18,19]. The MS conditions were as follows: First-order mass spectrometry parameters: Detector Type: Orbitrap, Resolution: 60,000, Cycle time (s): 3, RF lens (%): 30, AGC target: 4.0 × 10^5^, Microscans: 1, Data type: profile, Maximum IT: 50 ms and Scan range: 275–1500 [19]. Secondary mass spectrometry parameters: Detector Type: Orbitrap. Resolution: 15,000, first mass (m/z): 110, AGC target: 5.0 × 10^5^, Maximum IT: 256, microscans: 1, data type: centroid, HCD Collision Energy (%): 30 [16,17,18,19].

### 4.7. Data Processing

We analyzed all LC-MS/MS raw data files using Proteome Discoverer 2.2 software (version 2.2; Thermo Fisher Scientific). Difference screening criteria: Ratio > 1.5 or <0.67, *p*-value < 0.05 [9,19]. Due to insufficient protein annotations for goat species, we searched for data in a “Ruminantia” database from UniProt (protein database: ruminantia-filtered-reviewed_yes.fasta) [9,20]. Analysis settings: Enzyme Name, Trypsin (Full), Precursor Mass Tolerance, 20 ppm, Fragment Mass Tolerance, 0.5 Da, Static Modification: Carbamidomethyl/+ 57.021 Da(C). Processing node 1. Spectrum Properties Filter: Lower RT Limit, 0. Upper RT Limit, 0. Min. Precursor Mass, 350 Da. Max. Precursor Mass, 5000 Da. A maximum of two missed cleavages was allowed. The maximum false discovery rate for peptide and protein was specified as 0.01; the LFQ (Label-Free Quantitation) was enabled with the LFQ minimum ratio count to 1. Regression Settings: Regression Model, Non-linear Regression, Parameter Tuning, Coarse. The other settings were kept as default. GraphPad Prism v7.0 was used with all data pre-tested for normality [9,20]. Additionally, the values are presented as mean ± SD. Difference between an experimental group and a control was analyzed with Student’s *t*-test or one-way ANOVA [9,18]. The dataset identifier IPX0004443000. https://www.iprox.cn/page/PSV023.html;?url=1677679444384dvFy (accessed on 5 March 2023) [3,4,5]. Passport: OkPc. The mass spectrometry proteomics data have been deposited to the ProteomeXchange Consortium (http://proteomecentral.proteomexchange.org (accessed on 5 March 2023)) via the iProX partner repository with the dataset identifier IPX0004443000 [12,13,14].

### 4.8. Bioinformatics of Differentially Expressed Proteins

#### 4.8.1. Gene Ontology (GO) and Kyoto Encyclopedia of Genes and Genomes (KEGG) Annotations from Differentially Expressed Proteins

The Kyoto Encyclopedia of Genes and Genomes (KEGG) database (http://www.genome.jp/kegg/or http://www.kegg.jp/ (accessed on 22 July 2021)) was used to predict the main metabolic pathways. The protein sequences of differentially expressed proteins were retrieved in batches from the UniProtKB database (http://www.uniport.org/, 13 June 2017, release) in FASTA format [19,20]. The GO mapping and annotation (http://geneontology.org/ (accessed on 22 July 2021)) results were plotted using Cluster 3.0 and Blast2 GO (version 3.3.5). The GO annotation results were plotted using R scripts. Functional annotation was performed using the Gene Ontology (GO) database (http://www.geneontology.org (accessed on 22 July 2021)) and included the cellular component, molecular function, and biological process. Briefly, the identified protein ID was converted to UniProt ID and then mapped to GO IDs by protein ID. The retrieved credibility is ≥95%, false positive is <5% in the database; difference ratio of proteins is ≥1.5-fold and *p*-value is ≤0.05. The enrichment analysis revealed 20 significant GO terms by hypergeometric test (*p* < 0.05). The FASTA protein sequences of differentially expressed proteins (DEPs) were blasted against the online GO database (http://geneontology.org/ (accessed on 22 July 2021)) to retrieve their GO annotations and were subsequently mapped to pathways in KEGG. GO and KEGG pathway enrichment analyses were applied based on Fisher’s exact test. The LFQ was selected for the quantification. The hypothesis was performed using the *t*-Test, and the associated *p*-values were adjusted based on Benjamini–Hochberg’s correction (FDR < 0.05). Briefly, the Benjamini–Hochberg correction for multiple tests was further applied to adjust the derived *p*-values. Only functional categories and pathways with *p*-values under a threshold of 0.05 were considered significant. The other settings were kept as default [20,21]. The analysis showed the 20 most relevant pathways sorted by *p*-value. The pathway enrichment analysis was performed using the database for annotation, visualization and integrated discovery.

#### 4.8.2. Hierarchical Clustering from Differentially Expressed Proteins

The related protein data were used to perform hierarchical clustering analysis [20,22]. For this purpose, Cluster 3.0 (http://bonsai.hgc.jp/~mdehoon/software/cluster/software.htm (accessed on 22 July 2021)), Java TreeView software (http://jtreeview.sourceforge.net (accessed on 22 July 2021)) and R scripts were used. The Euclidean distance algorithm for similarity measurement and the average linkage clustering algorithm (clustering using the centroids of the observations) were selected during hierarchical clustering [22,23].

#### 4.8.3. Protein–protein Interaction (PPI) Networks from Differentially Expressed Proteins

The PPI information for the studied proteins used the gene symbols and STRING software, version 11 [23]. The PPI information for the studied proteins was retrieved from the IntAct molecular interaction database (http://www.ebi.ac.uk/intact/ (accessed on 22 July 2021)) using the gene symbols and STRING (http://string-db.org/ (accessed on 22 July 2021)) software. The difference ratio of proteins is ≥1.5-fold and *p*-value is ≤0.05; identified proteins must be redundant by all artificial. The minimum interaction score was set to medium at 0.40 as the confidence score. The primary default setting was used for the analysis. The results were downloaded in XGMML format and imported into Cytoscape5 software (http://www.cytoscape.org/ (accessed on 22 July 2021), version 3.2.1) to visualize and further analyze the functional PPI networks [23,24].

### 4.9. Transmission Electron Microscopy (TEM)

The cell was fixed with 2% glutaral dehyde, 2% paraformaldehyde buffered with 0.1 M phosphate buffer, pH 7.2 overnight at 4 °C, followed by post-fixation with 1% osmium tetroxide in a 0.1 M sodium cacodylate buffer, pH 7.4 for 1.0 h at room temperature, and graded ethanol dehydration [15,16]. Samples were embedded in Epon (Electron Microscopy Sciences, Shinagawa-ku, Tokyo, Japan) and sectioned using an ultramicrotome (Leica, Germany) [16,17].

### 4.10. qRT-PCR

Solation of total RNA from the gEECs cultures was performed using Trizol reagent (Invitrogen, Carlsbad, CA, USA) according to the manufacturer’s protocol [10,25]. Total RNA (500 ng) was reverse-transcribed into a cDNA template using PrimeScript RT Kit with gDNA Eraser (TaKaRa, Dalian, China). Quantitative Real-time PCR was performed using SYBR Premix Ex Taq (TaKaRa, Dalian, China) on iQ5 thermal cycler (Bio-Rad, San Francisco, CA, USA) [2,5,6]. The mRNA expression levels of IL-6 and TNF-α were measured relative to the GAPDH reference gene using the 2^−ΔΔCt^ method. The details of the primers were: IL-1β, XM_013967700.2 (Forward: TCCACCTCCTCTCACAGGAAA, Reverse: TACCCAAGGCCACAGGAATCT), IL-6, NM_00128564.1 (Forward: CCTCTTCACAAGCGCCTTCA, Reverse: TGCTTGGGGTGGTGTCATTC) and GAPDH, XM_005680968.3 (Forward: GATGGTGAAGGTCGGAGTGAAC, Reverse: GTCATTGATGGCAACGATGT). The mRNA expression levels were calculated using the 2^−ΔΔCt^ method.

### 4.11. Western Blot

Equivalent amounts of protein (15.0 μg) were loaded on 12% SDS-PAGE gels and then transferred onto 0.45 μm nitrocellulose membrane (Millipore; Bedford, MA, USA) [19,20]. The samples were incubated with anti-COL1A1 (#P28373; Abmart; diluted 1:2000), anti-p-AKT (Ser473, #4060; CST; diluted 1:2000) and anti-SERPINE1(#YT-3569; Immunoway, Plano, TX, USA) [21,22]. The membranes were incubated for 2.5 h with horseshoe radish peroxidase-labeled secondary antibody (ZHONGHUIHECAI, Xi’an, China) at room temperature. Detection was performed (Dining, Beijing, China) and recorded by film exposure [22,23,24].

### 4.12. Confocal Microscopy

Cells were seeded in confocal dishes until growth was >80%. The cell culture medium was removed by aspiration and cells were washed once with PBS. The treatment was performed as described. The cells were fixed by 4% paraformaldehyde for 20 min, then permeabilized with 0.5% triton-x diluted for 20 min. The cells were blocked with 10% FBS in 1× PBS containing 0.5% Tween-20 [14,15].

The primary antibodies (anti-COL1A1; diluted 1:200, anti-KNG1; diluted 1:200 and anti-SERPINE1; diluted 1:200) were added and the cells at a concentration were incubated overnight at 4 °C. The following day, the cells were washed and added to the secondary antibodies (Alexa-labeled donkey anti-rabbit IgG (Invitrogen, Life Technologies, Carlsbad, CA, USA; diluted 1:500)) for 2 h at room temperature in the dark [20,26]. They were dyed by DAPI (C1005, Beyotime Biotechnology, Shanghai, China) at 37 °C for 10 min. The cells were mounted by ProLong Glass Antifade Mountant (Antifade Mounting Medium, Beyotime Biotechnology). The cells were observed by a Nikon A1+/A1R+ microscope (30 × objective, Nikon Inc., Melville, NY, USA) [9,20,21,22,23]. The anti-COL1A1 (TU-425068) and anti-KNG1 (TU-418934) were purchased from Abmart antibodies, ShangHai, China [27]. The anti-SERPINE1 (YT-3569) was purchased from Immunoway, Plano, TX,75024 USA [21,22,23,24].

## 5. Conclusions

One of the most popular techniques for finding potential biomarkers for many diseases in recent years is quantitative methods of mass, which include ultra-sensitive mass spectrometry. A total of 1180 proteins were identified in the goat Endometrial Epithelial Cells and LPS-treated goat Endometrial Epithelial Cell groups, of which 313 differential proteins were accurately screened. Compared with the goat Endometrial Epithelial Cells group, the inflammatory response, endoplasmic reticulum stress, apoptosis and mitochondrial function were activated or inhibited from differentially expressed proteins in the LPS-treated goat Endometrial Epithelial Cells group. The results of differentially expressed proteins showed that several signaling pathways related to biological processes or molecular function of proteins were upregulated or downregulated. The results were independently verified by WB, TEM and IF techniques, and the results were consistent with the proteomic data. Additionally, some of these proteins can be used as biomarker proteins to predict diseases. Our team will continue to study the relevant molecular mechanisms in depth. All findings reported here may provide new ideas and directions to improve our understanding of the biological mechanism of endometritis.

## Data Availability

The mass spectrometry proteomics data have been deposited to the ProteomeXchange Consortium (http://proteomecentral.proteomexchange.org (accessed on 5 March 2023)) via the iProX partner repository with the dataset identifier IPX0004443000, https://www.iprox.cn/page/MSV022.html (accessed on 5 March 2023). “The datasets presented in this study can be found in online repositories. The name of the repository and accession number can be found below: iProX (https://www.iprox.cn/ (accessed on 5 March 2023)); IPX0004443000.” The datasets generated and/or analysed during the current study are available in the (Uniprot) repository, (COL1A1, Protein ID: P02453; SERPINE1, Protein ID: P13909; KNG1, Protein ID: P01044). The data that supports the findings of this study are available in the supplementary material of this article. “Not applicable” for studies not involving humans or animals.

## Supplementary Materials

The supporting information can be downloaded at: https://www.mdpi.com/article/10.3390/ijms241210018/s1.
